# Energy Balanced Strategies for Maximizing the Lifetime of Sparsely Deployed Underwater Acoustic Sensor Networks

**DOI:** 10.3390/s90906626

**Published:** 2009-08-24

**Authors:** Hanjiang Luo, Zhongwen Guo, Kaishun Wu, Feng Hong, Yuan Feng

**Affiliations:** 1 Department of Computer Science, Ocean University of China, Qingdao, China; E-Mails: guozhw@ouc.edu.cn (Z.-W.G.); hongfeng@ouc.edu.cn (H.F.); fengyuan@ouc.edu.cn (Y.F.); 2 Department of Computer Science & Engineering, Hong Kong University of Science and Technology, Hong Kong, China; E-Mail: kwinson@cse.ust.hk (K.-S.W.)

**Keywords:** acoustic communications, energy efficiency, underwater acoustic sensor networks, data propagation, energy balancing

## Abstract

Underwater acoustic sensor networks (UWA-SNs) are envisioned to perform monitoring tasks over the large portion of the world covered by oceans. Due to economics and the large area of the ocean, UWA-SNs are mainly sparsely deployed networks nowadays. The limited battery resources is a big challenge for the deployment of such long-term sensor networks. Unbalanced battery energy consumption will lead to early energy depletion of nodes, which partitions the whole networks and impairs the integrity of the monitoring datasets or even results in the collapse of the entire networks. On the contrary, balanced energy dissipation of nodes can prolong the lifetime of such networks. In this paper, we focus on the energy balance dissipation problem of two types of sparsely deployed UWA-SNs: underwater moored monitoring systems and sparsely deployed two-dimensional UWA-SNs. We first analyze the reasons of unbalanced energy consumption in such networks, then we propose two energy balanced strategies to maximize the lifetime of networks both in shallow and deep water. Finally, we evaluate our methods by simulations and the results show that the two strategies can achieve balanced energy consumption per node while at the same time prolong the networks lifetime.

## 1. Introduction

Underwater Acoustic Sensor Networks (UWA-SNs) have recently been drawing much attention because of their potential applications ranging from oceanographic data collection, environment monitoring, structure monitoring, tactical surveillance to disaster prevention [1, 2]. However, UWA-SNs are very different from existing terrestrial sensor networks due to the properties of the underwater environments. Firstly, UWA-SNs use acoustic signals to communicate, thus the propagation delay is large due to the slow acoustic signal propagation speed (1.5 × 10^3^*m/s*). Secondly, the underwater acoustic communication channel has limited bandwidth capacity because of the significant frequency and distance dependent attenuation. Currently, the limit on available underwater bandwidth is roughly 40 km×kbps [3, 4]. Thirdly, due to economics and the potentially large areas of interest in the ocean, UWA-SNs are mainly sparse networks nowadays [2, 3]. For such networks, instead of randomly deploying the sensor nodes, it is common to deploy the nodes manually with help of ships [5].

To deploy such a long-term UWA-SNs, one of the main challenges is the limited energy resources of the sensors because they are battery-powered and it is even harder to recharge node batteries in underwater environments. With such sparsely deployed networks, the energy balanced dissipation of individual nodes become very important, and it is desirable to distribute energy consumption of each node evenly, thus nodes may die together and we can change their batteries or replace them by new nodes simultaneously for the whole area.

However, in such sensor networks, sensed data are usually routed to sinks and such centralized data transmissions lead to unbalanced energy consumption which refers to as the “energy hole” problem. This may partition the whole networks and make sub-regions uncovered or even collapse the entire networks [6].

The “energy hole” problem has been well studied in terrestrial sensor networks [6–9]. However, UWA-SNs have unique characteristics very different from terrestrial sensor networks, and these algorithms can not be directly applied to the UWA-SNs.

First, the energy consumption of acoustic modem in UWA-SNs is quite different from those of typical radio transceivers, for the transmit power is dependent on both distance and frequency. Furthermore, transmit power is often 100 times more expensive than that of the receive mode in UWA-SNs [10, 11]. For example, the typical receive power of the WHOI micro-modem is about 80 mW, but the transmit power is 10 W [12].

Second, the algorithms in terrestrial networks use dense network model and mainly try to achieve energy balance among different clusters or slices, without considering energy balance of individual nodes [13, 14]. However, in UWA-SNs, node deployment is generally sparse because of economic considerations [3]. Therefore, the energy balance of individual nodes become very important in such networks.

Several literatures address the energy consumption in UWA-SNs. In [15], the author estimates the battery lifetime and power costs of shallow water in terms of four independent parameters: distance, frequency, frequency of data updates and number of nodes in cluster. In [16], the author analyzes the energy consumption in UWA-SNs considering routing protocols such as packet relaying, direct transmission and clustering in both shallow and deep water. However, both [15] and [16] just consider the total energy consumption of networks, and do not take the energy balance of individual nodes into account. Though the total energy consumption is a significant energy efficiency metric, the energy consumption of individual node is more important for sparsely deployed UWA-SNs because the death of a single node in such networks may cause the networks to become disconnected or collapse the entire networks [17].

In this paper, we focus on the energy balance problem and try to achieve energy balance among individual nodes considering two types of sparsely deployed UWA-SNs: underwater moored monitoring systems and sparsely deployed two-dimensional UWA-SNs. We first analyze the reasons of unbalanced energy consumption in such networks, then we propose two different energy balanced strategies: Energy Balanced Hybrid (EBH) data propagation algorithm and Differential Initial Battery (DIB) assignment to achieve balanced energy consumption of individual nodes. EBH is a hybrid data propagation algorithm which alternately changes node’s transmit mode between multi-hop and direct transmission based on node’s residual energy gradation to achieve energy balance among sensor nodes. DIB takes another strategy to deal with the unbalanced energy consumption problem, in which it pre-assigns differential battery power level according to node’s traffic loads before deployment in two-tier hierarchical networks.

We evaluate the proposed strategies extensively by simulations and the results show both EBH and DIB can achieve balanced energy consumption per-node, and at the same time maximize the lifetime of such networks in both shallow and deep water.

The main contributions of our work are:

1. We have theoretically analyzed the energy balanced consumption of individual nodes in a linear sensor network for both shallow and deep water.
2. We proposed two different energy balanced strategies: EBH and DIB to maximize the lifetime of sparsely deployed UWA-SNs.

The rest of this paper is organized as follows. Section 2 describes the network model and underwater acoustic propagation. Section 3 analyzes the reasons of unbalanced energy consumption in linear sensor networks. In Section 4, we present the energy balance strategies in detail. Simulations are described in Section 5. And the related work is presented in Section 6. The conclusions and future work are discussed in Section 7.

## 2. Network Model and Underwater Acoustic Propagation

In this section, we first present our network model, then we review underwater acoustic propagation.

### 2.1. Network model

We first consider a sparsely deployed UWA-SNs: an underwater moored monitoring system in this paper. Underwater moored monitoring systems provide unprecedented abilities and opportunities to monitor changes in oceans and atmosphere by collecting real-time sensed data throughout the entire water column over large temporal scales [18]. These real time oceanographic data enable us to better understand the oceans, and to solve world problems such as natural disaster prediction and global warming.

**Figure 1.**
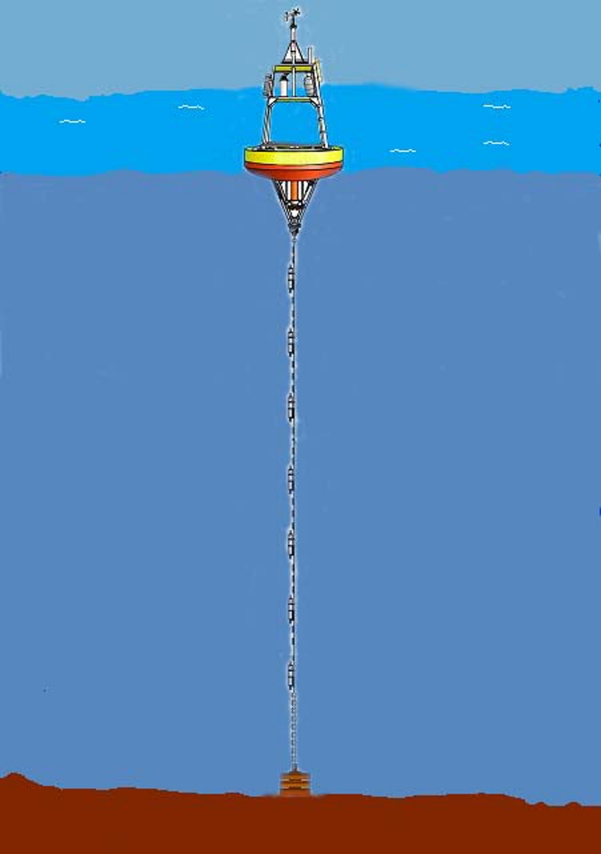
Underwater acoustic moored monitoring system.

As shown in Figure 1, a typical moored oceanic monitoring system is composed of an anchor, a mooring line, and a floating buoy (surface sink node) with satellite, radio frequency (RF) or cell phone technology to transmit data to shore in real-time [19]. The sensors are attached to the mooring line and they may be physical, hydrographic, bio-optical, or chemical sensors which can measure temperature, conductivity, salinity, pressure, current speed etc. The periodic sensed data must be transmitted from different depths in the water column to the surface buoy.

Though these sensors can be wired together using special mooring cable, the wired system has some disadvantages, such as cable breakage, too expensive to be widely used, etc. [19]. It is cost-effective and robust alternatives to use wireless acoustic communication in such mooring systems. In that case, these sensors form a linear acoustic sensor network.

There are mainly two types of applications of UWA-SNs: event-driven and periodic sensing, which lead to different traffic patterns [17]. In this paper, we focus on periodic sensing networks in which the nodes constantly sense the environment and report their findings to an end node. The moored monitoring system is working in periodic sensing. To evaluate the lifetime of the networks, we divide the time into rounds as it has been done in most previous research [7]. For simplicity, we assume each node precisely generates one data packet in each round.

### 2.2. Underwater acoustic propagation

In this section, we review the energy consumption characteristics of a typical acoustic modem.

#### The passive sonar equation

The passive sonar equation [22] expresses the signal to noise ratio (SNR) of an emitted underwater signal at the receiver:

(1) SNR=SL−TL−NL+DI≥DT

where SL is the source level, TL is the transmission loss, NL is the noise level, DI is the directive index, and DT is the detection threshold of the sonar. The quantities of the Equation (1) are in *dB re μPa*, where the reference value of 1 *μPa* equals to 0.67 × 10^−18^ *Watts/m*^2^ [22]. We use the notation *dB* to signify *dB re μPa* in the rest of this paper.

The noise level in shallow water is related to shipping activity, wind level, biological noise, seaquakes etc. In this paper, we take the value of the NL to be 70 dB in shallow water [22]. The deep sea is more quiet than shallow water, and we consider NL to be 50 dB [24]. DI and SNR are related to acoustic modems and hydrophones, we take DI = 3 dB and SNR = 20 dB respectively [16].

#### Transmission loss

The acoustic signals propagate differently in shallow water and deep sea. The shallow water refers to water with depth lower than 100 m [16]. In shallow water, acoustic signals propagate within a cylinder bounded by the sea surface and sea floor [25]. The transmission loss for shallow water can be expressed as [26]:

(2) $$\mathrm{TL}=10\;\text{log}\;d+\alpha d\times 10^{-3}$$

where *d* is the distance between source and receiver expressed in meters, *α* is the absorption coefficient with the unit *dB/km*, and *T L* is in *dB*.

In deep sea, transmission loss is caused by spherical spreading and absorption. The loss can be expressed as [26]:

(3) $$\mathrm{TL}=20\;\text{log}\;d+\alpha d\times 10^{-3}$$

in *dB*.

Equations (2) and (3) indicate that transmission loss are mainly caused by distance dependent attenuation and frequency dependent absorption both in shallow water and deep sea.

The frequency dependent absorption coefficient *α* is calculated by Thorp’s expression in [22] for frequencies above a few hundred Hertz as:

(4) $$\alpha =\frac{0.1\;f^{2}}{1+f^{2}}+\frac{40\;f^{2}}{4100+f^{2}}+2.75\times 10^{-4}\;f^{2}+0.003$$

where *α* is in *dB/km* and *f* is the frequency in kHz.

For lower frequencies, *α* is expressed as:

(5) $$\alpha =0.11\frac{f^{2}}{1+f^{2}}+0.011\;f^{2}+0.002$$

where *α* is in *dB/km* and *f* is the frequency in kHz.

#### Transmission power

The source level SL is related to the transmitted signal intensity at 1 m from the source which can be expressed as:

(6) $$\mathrm{SL}=10\;\text{log}\;\frac{I_{T}}{1\mu \mathrm{Pa}}$$

where *I_T_* is in *μPa*. From Equation (6) we obtain *I_T_* in *Watts/m*^2^:

(7) $$I_{T}=10^{\mathrm{SL}/10}\times 0.67\times 10^{-18}.$$

For shallow water, to achieve an intensity *I_T_* at a distance of 1 m from the source, the source transmitter power *P_T_*(*d*) should be:

(8) $$P_{T}\;(d)=2\pi \times 1m\times H\times I_{T}$$

in *Watts/m*^2^, where *H* is the sea depth in *m*.

For deep sea, the source transmitter power *P_T_*(*d*) should be:

(9) $$P_{T}\;(d)=4\pi \times {\left(1m\right)}^{2}\times I_{T}$$

in *Watts/m*^2^.

Now we can calculate the required transmit power for signal transmissions at a given distance *d* and frequency *f*.

Through Equation (1), we represent source level SL as:

(10) SL=SNR+TL+NL−DI

where TL can be calculated via Equation (2) and (3) in shallow water or in deep sea. Then through Equation (7), we obtain source intensity *I_T_*. Finally, using Equations (8) and (9), we obtain the required transmit power *P_T_* in shallow water or deep sea.

For shallow water,

(11) $$P_{T}\;(d)=P_{T-\mathrm{shallow}}\;(d)=2\pi H\times 10^{\mathrm{SL}/10}\times 0.67\times 10^{-18}$$

For deep sea,

(12) $$P_{T}\;(d)=P_{T-\mathrm{deep}}\;(d)=4\pi \times 10^{\mathrm{SL}/10}\times 0.67\times 10^{-18}$$

## 3. The Reasons of the Unbalanced Energy Consumption

We analyze the energy consumption of the linear network underwater in this section.

**Figure 2.**
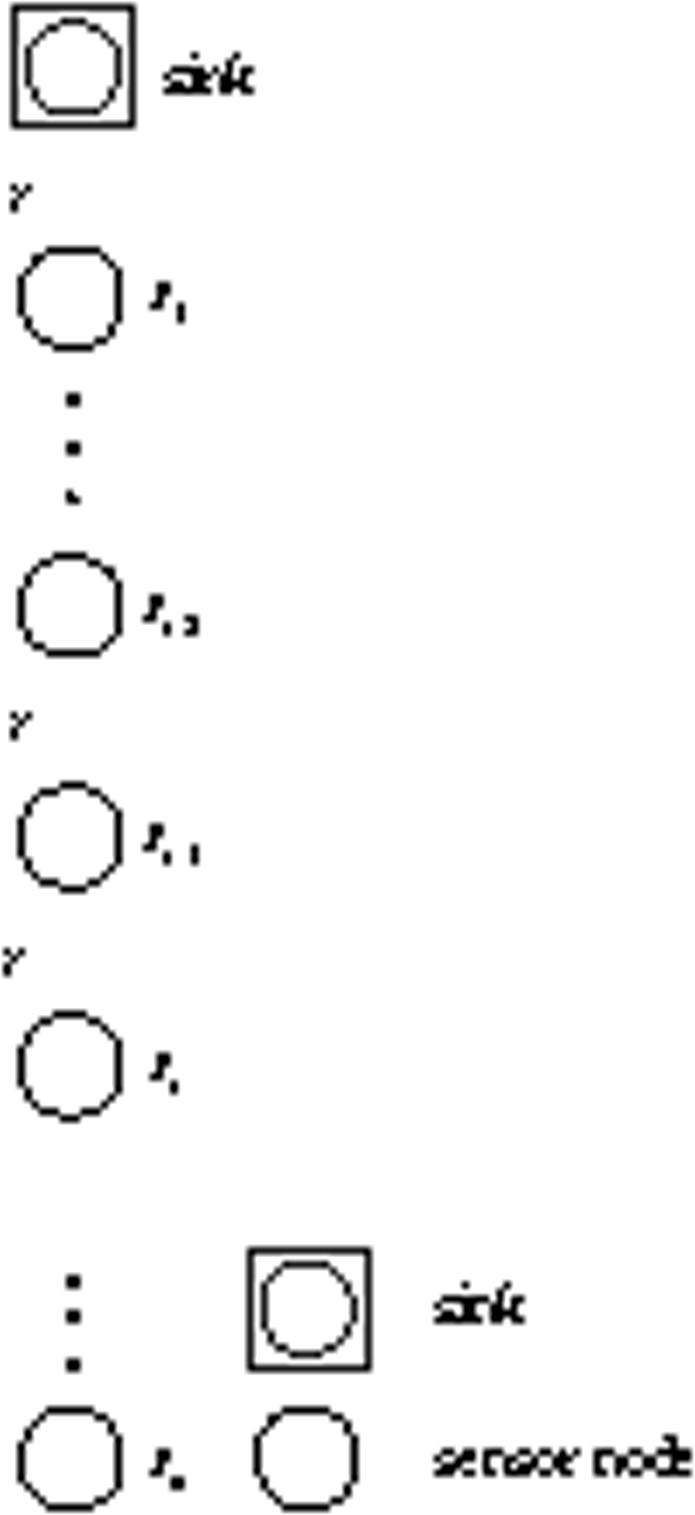
Linear sensor network.

We assume sensor nodes as *S* = {*S_i_*|*i* ∈ {1,......, *n*}} form the linear sensor network, just as Figure 2 shows. For analytical simplicity, we assume the internode distance between two adjacent nodes is *r*. As to ∀*S_i_* ∈ *S*(*i* = 3,......, *n*), *S*_*i*−2_, *S*_*i*−1_ and *S_i_* are adjacent nodes in the linear network where *S*_*i*−2_ is closer to the sink. *S*_*i*−1_ has two neighbors *S_i_*, *S*_*i*−2_, in which we define *S_i_* as the upstream neighbor of *S*_*i*−1_ and *S*_*i*−2_ as the downstream neighbor of *S*_*i*−1_.

Specifically, let’s consider a common scenario: each node in the chain has a packet with *k* bits message, such as temperature, pressure, salinity, etc., to send to the sink in each round. Data aggregation techniques may reduce packet transmissions, especially in large sensor networks where data generated from neighboring sensors are always highly redundant and correlated [23]. However, in sparsely deployed UWA-SNs, not much data redundancy can be exploited and the data may not be correlated. For example, the physical or chemical monitored parameters such as temperature, conductivity, salinity, pressure, and current speed of ocean are not correlated. Thus, we consider applications that data aggregation cannot reduce much of the data traffic.

We first consider each sensor sends the packet hop by hop to the sink via the linear sensor network which we denoted as HBH. The energy consumption for the nodes in the chain is different. Considering two adjacent nodes: *S_i_* and *S*_*i*−1_, *S*_*i*−1_ not only transmits its own sensed data towards the sink, but also relays the data it receives from *S_i_*. Thus during the same period of time, *S*_*i*−1_ has more packets to send than *S_i_*, which leads to uneven energy consumption. The energy consumption ratio of *S*_1_ to *S_n_* is *N* in each round. As a result, the nodes near the sink will deplete energy more quickly and eventually die out first.

Now, we consider each node in the linear network sends sensed data directly to the sink, which we denoted as DIRECT. Equations (2) and (3) show that transmission losses in both shallow water and deep sea are related to distance, which means that the nodes far from the sink deplete energy more quickly and eventually die out first.

Summarizing hop by hop and direct transmission, the unbalanced energy consumption problem is caused mainly by the different distances to the sink and the way of data transmission. To solve the uneven energy consumption problem, we propose two different strategies to achieve balanced energy consumption between nodes, and at the same time maximize the lifetime of such networks both in shallow water and deep sea.

## 4. Energy Balanced Strategies for Sparsely Deployed UWA-SNs

In this section, we first present two energy balanced strategies in detail, then we discuss extending the linear network algorithms to two-dimensional UWA-SNs.

### 4.1. EBH: an energy balanced hybrid data propagation algorithm

We propose a hybrid data propagation algorithm EBH to achieve balanced energy consumption of individual nodes. The key insight of the algorithm is to combine the advantages of two data transmission methods.

The sensor nodes are equipped with transmission power control module which has adjustable transmission power [24, 27, 28]. The node may transmit data via multi-hop route to the sink, which we defined as *MODE*0 or directly to the sink with suitable transmission power, which we defined as *MODE*1. Therefore, the nodes use a hybrid transmission strategy for data propagation, which is the core design of EBH. By alternately changing node’s working mode based on the residual energy grade, the nodes dissipate energy evenly, so the lifetime of the network can be prolonged.

We first overview the algorithm, and then we compute the optimum number of energy grade in terms of maximizing the network lifetime. At the end of this section, we present the practical algorithm in detail.

#### Overview of EBH

**Figure 3.**
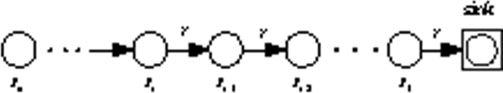
Linked sensor network.

**Figure 4.**
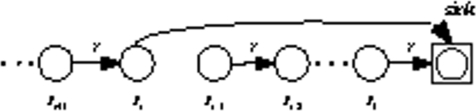
Unlinked sensors.

We divide the node’s initial energy into *m* units and each unit denotes as one gradation of energy. Initially, each node works in *MODE*0 and the linear sensor network is a multi-hop network. The nodes closer to the sink will dissipate more energy. Let’s again consider two adjacent nodes: *S_i_*, *S*_*i*−1_ in Figure 3. *S*_*i*−1_ first consumes one gradation of energy and drops into the next energy gradation. Then it sends a control message which includes its energy information to upstream node *S_i_*. When the upstream node *S_i_* receives the control message, it compares its own grade number with the grade number in the control message. If the grade number is higher than the downstream node’s, it changes working mode to *MODE*1. Just as Figure 4 shows, *S_i_* will transmit data directly to the sink, which mitigates its downstream node’s loads. Otherwise, it relays the control message to upstream node until the message finds a node which has higher energy. In the same way, when *S_i_* has consumed one gradation of energy, it does the same thing as *S*_*i*−1_ and changes back to *MODE*0. In other words, it links to *S*_*i*−1_ again just as Figure 3 shows. By alternatively changing node’s transmit mode between multi-hop and direct transmission based on residual energy, the nodes in the linear network dissipate energy evenly, so the lifetime of the network prolongs.

#### Optimum gradation number of linear network

In EBH, when node’s residual energy gradation changes, the node changes the working mode. However, how many energy grades are required to maximize the network lifetime? On one hand, if the number of grades *m* is too small, EBH tends to be a multi-hop transmission scheme which leads to uneven energy consumption. On the other hand, if the number of grades *m* is too large, the transmission mode of nodes will alternate frequently between *MODE*0 and *MODE*1, which incurs a large amount of control messages and consumes tremendous energy. Therefore, finding the appropriate energy gradations of node is very important. Next we calculate the appropriate grades *m*.

We assume the initial energy of each sensor is *E* > 0, and the nodes implement optimal sleep schedule protocol, in which the nodes are in sleep mode and only wake up during active transmission and reception [17]. The total energy consumption in each round includes transmitting, receiving and sensing.

The total energy grades denote as *m*, then one gradation of energy is *E/m*. We notice that the farthest node from the sink consumes more energy when transporting data directly to the sink. Just as Figure 2 shows the node *S_n_* is the farthest node from the sink. Let *E_d_* be the energy dissipation that the farthest node spent on transmitting one packet directly to the sink and *E_TX_* (*k, nr*) be the energy dissipation of sending a *k* bits packet over distance *nr*. Then we have

(13) $$E_{d}=E_{\mathrm{TX}}\;(k,\;\mathrm{nr})=P_{T}\;(\mathrm{nr})\times T_{\mathrm{TX}}$$

where *E_d_* is in *Joule*, *P_T_* (*nr*) is the transmit power which can reach distance *nr*, *T_TX_* is the duration of transmit time in second.

If *E_d_* is less than one gradation of energy, then we have:

(14) $$E_{d}<E/m.$$

We use Equation (14) as a condition to calculate *m*. The reason is that if the farthest node from the sink consumes less than one unit energy, then the other nodes also consume less than one unit energy when transmitting one packet directly to the sink. Therefore, any two adjacent nodes at most differ only one unit of energy. Next, we give our proof in Lemma 1.

We first give some definitions to deduce Lemma 1. As shown in Figure 3, the distance between *S*_*i*−1_ and the sink is *D*, then *D* = (*i* − 1)*r*. Thus the distance between *S_i_* and the sink is *D* + *r* and *D* + *r* = *ir*.

Let *RG*(*s_i_*) be the residual energy gradations of node *S_i_*. Let *E_a_*(*s_i_, t*) be the total energy dissipation of node *S_i_* during time period of *t* rounds.

**Lemma 1:** If *E_d_* < *E/m*, then *RG*(*s_i_*) − *RG*(*s*_*i*−1_) ≤ 1.

**Proof :** Because *E_d_* < *E/m*, then any node transmitting one packet will dissipate less than one unit of energy.

(1) We first consider the initial deployment. Each node is working in *MODE*0, and forms a multi-hop network. We consider ∀*S_i_* ∈ *S*(*i* = 2,......, *n*), *S*_*i*−1_ and *S_i_* are adjacent nodes in the linear network where *S*_*i*−1_ is closer to the sink, just as Figure 3 shows. *S*_*i*−1_ not only transmits its own sensed data but also relays *S_i_*’s packets. So *S*_*i*−1_ has more packets to transmit than *S_i_* during the same period of *t* rounds. We denote *E_SEN_* (*k*) as the energy consumption of sensing *k* bits message and *E_RX_*(*k*) as the energy dissipation of receiving a *k* bits packet.

Then, we have

$$\begin{array}{lll} E_{a}\;\left(s_{i},\;t\right) & = & t\times E_{\mathrm{SEN}}\;(k)+u\times \\ & & \left(E_{\mathrm{RX}}\;(k)+E_{\mathrm{TX}}\;(k,\;r)\right)+t\times E_{\mathrm{TX}}\;(k,\;r)\\ E_{a}\;\left(s_{i-1},\;t\right) & = & t\times E_{\mathrm{SEN}}\;(k)+(u+1)\times \\ & & \left(E_{\mathrm{RX}}\;(k)+E_{\mathrm{TX}}\;(k,\;r)\right)+t\times E_{\mathrm{TX}}\;(k,\;r) \end{array}$$

where *u* is the number of packets relayed by *S_i_* during *t* rounds. Thus *S*_*i*−1_ dissipates energy more quickly than *S_i_*. As a result, *S*_*i*−1_ will first consume one gradation of energy and drop into the next energy grade. Then, it sends out a control message to *S_i_*. At that time, *RG*(*s_i_*_1_) − *RG*(*s*_*i*−1_) ≤ 1.

(2) When *S_i_* receives the control message sent by *S*_*i*−1_, it changes working mode to *MODE*1 because *S_i_* has higher grade number than *S*_*i*−1_, thus it sends data directly to the sink just as Figure 4 shows. We denote the time duration as *v* rounds before *S_i_* dropping into the next energy grade. We obtain

$$\begin{array}{lll} E_{a}\;\left(s_{i},\;v\right) & = & v\times E_{\mathrm{SEN}}\;(k)+w\times E_{\mathrm{RX}}\;(k)\\ & & +(w+v)\times E_{\mathrm{TX}}\;(k,\;D+r)\\ E_{a}\;\left(s_{i-1},\;v\right) & = & v\times \left(E_{\mathrm{SEN}}\;(k)+E_{\mathrm{TX}}\;(k,\;r)\right) \end{array}$$

where *w* is the number of packets relayed by *S_i_* during the time period of *v* rounds. Because *E_a_*(*s_i_, v*) ≫ *E_a_*(*s*_*i*−1_, *v*), before *S_i_* dropping into the next energy grade and having the same grade with *S*_*i*−1_, the energy difference between them is diminishing, i.e., *RG*(*s*_*i*−1_) − *RG*(*s_i_*) < 1.

(3) When *S_i_* dropping into the next energy grade, it has the same grade with *S*_*i*−1_. We have *RG*(*s_i_*) *− RG*(*s_i−_*_1_) = 0.

The result was proved.

Next, using the **Lemma 1**, we have **Theorem 1**.

**Theorem 1**: If *E_d_ < E/m*, the optimum grades for linear network is given by $m=\min\left(\sqrt{\frac{\mathrm{nE}}{{2E}_{C}},\;\frac{E}{E_{d}}}\right)$.

**Proof:** Let *E_C_* be the energy spent on transmitting one control message to inform upstream node about its residual energy. We have

$$E_{C}=E_{\mathrm{TX}}\;(c,\;r)$$

where *c* is the bits of one control message. Let *E_waste_*(*m*) be the wasted energy which includes both the residual energy when the network collapses and the energy spent on sending control messages. Depending on different applications and the network lifetime definitions, when the network collapses, there are at most *n* − 1 nodes still alive. From **Lemma 1**, we know two adjacent nodes’ residual energy at most differ one gradation of energy. Thus, we obtain

$$E_{\mathrm{waste}}\;(m)=\sum_{i=1}^{n-1}i\times \frac{E}{m}+(n-1){\mathrm{mE}}_{C}\;\;\;\left(E_{d}<E/m\right).$$

The derivative of *E_waste_*(*m*) is

$$E_{\mathrm{waste}}^{\prime }\;(m)=\frac{n(n-1)}{2}\times \frac{-E}{m^{2}}+(n-1)E_{C}.$$

Let *E′_waste_*(*m*) = 0 then we have $m=\sqrt{\frac{\mathrm{nE}}{{2E}_{C}}}$, (*E_d_* < *E*/*m*).

Thus, the optimum grade for linear network is

(15) $$m=\min\left(\sqrt{\frac{\mathrm{nE}}{{2E}_{C}},\;}\frac{E}{E_{d}}\right).$$

#### EBH algorithm

To make EBH practical, we should take other things into consideration before we present the algorithm in detail.

First, how to set up the linear network in the initialization phase? It is easy to find node’s neighbors and at the same time to set up the linear network. For example, at the initialization of the network, the sink can broadcast a neighbor-finding control message to its one hop neighbor. When the node nearest to the sink receives the control message, it answers the sink with ACK message. Then it relays the control message to its one hop neighbor. By the same way, the control message is forwarded hop by hop along the linear network until it reaches the end node in the chain. The end node will not receive any ACK message after it relays the control message.

Second, what happens if one node dies suddenly due to reasons other than energy depletion? It depends on specific applications: some applications may demand that all nodes should alive in order to get the integrity of the whole datasets. Under such cases, when one node dies in the chain, the lifetime of network is over. Of course, some applications may survive the death of some nodes. In that case, we can change our algorithm a little bit to adjust to the new situation. For example, when a node dies suddenly, we can just let the dead node’s upstream neighbor fix on *MODE*1. In that way, the chain turns into two smaller chains and these chains will maintain energy balance.

Third, what is the impact of the overhead costs, such as the linear network maintenance? Because the control message is always much shorter compared with the normal sensed data and the transmit power is often 100 times more expensive than that of the receiving, the major part of energy consumption underwater is the transmission energy consumption. In addition, the overheads will consume energy and affect the residual energy of node. So, if the overheads lead to uneven energy consumption among nodes, the algorithm will make the nodes which have higher energy relay more data packets and eventually make the whole chain evenly, because the basic idea behind the algorithm is that the node with higher residual energy should do more work.

Now, we show EBH in **Algorithm 1**. At initialization phase, each node sets working mode to *MODE*0 and computes the unit of energy using the optimum grades *m*. The sink takes charge of setting up the linear networks. It broadcasts a neighbor-finding message to its one hop neighbor. After receiving the neighbor-finding message, the node records its downstream neighbor. Then it answers the sender with an ACK message including its own ID. After that, it relays the neighbor-finding message including its own id to the next node in the chain. When the downstream node receives the ACK message, it records the upstream neighbor.

**Algorithm 1:** EBH

1:	**procedure** NodeInitialization
2:	*Mode ← MODE*0
3:	*EnergyUnit ← E/m*
4:	*ResidualEnergyGradeNumber ← m*
5:	**return** TRUE
6:	**end procedure**
7:	**procedure** NeighborFindingMessageReceived
8:	*DownStreamNeighbor* = *NeighborFindingMessage.id*
9:	*SendNeighborFindingACK.id* = *idOfItself*
10:	*SendNeighborF indingACK*()
11:	*NeighborFindingMessage.id* = *idOfItself*
12:	*SendNeighborFindingMessage*()
13:	**return** TRUE
14:	**end procedure**
15:	**procedure** SendNeighborFindingACKReceived
16:	*UpStreamNeighbor* = *SendNeighborFindingACK.id*
17:	**return** TRUE
18:	**end procedure**
19:	**procedure** OneUnitEnergyConsumed
20:	*SendControlMessage*()
21:	**if***Mode* = *MODE*1 **then**
22:	*Mode* = *MODE*0
23:	**end if**
24:	**return** TRUE
25:	**end procedure**
26:	**procedure** ControlMessageReceived
27:	**if***ResidualEnergyGradeN > ControlMessage.ResidualEnergyGradeN* and *Mode* = *MODE*0 **then**
28:	*Mode* = *MODE*1
29:	**else**
30:	*SendControlMessage*()
31:	**end if**
32:	**return** TRUE
33:	**end procedure**
34:	**procedure** SendControlMessage
35:	*SendResidualEnergyNumber*()
36:	**return** TRUE
37:	**end procedure**

When a node has consumed one gradation of energy, it sends out a control message containing its residual energy to the upstream neighbor, and then it checks whether its mode is *MODE*1. If so, it changes to *MODE*0. When a node receives a control message from downstream node, it compares residual energy of itself with the information contained in the control message and decides whether it changes working mode or relays the control message. If it has higher residual energy than the downstream neighbor then it sets working mode to *MODE*1. Otherwise, it just sends out the control message to the upstream neighbor.

### 4.2. DIB: differential initial battery assignment strategy

In this section, we try to pre-assign differential initial battery power according to workloads in a two-tier hierarchical UWA-SNs to achieve balanced energy consumption.

**Figure 5.**
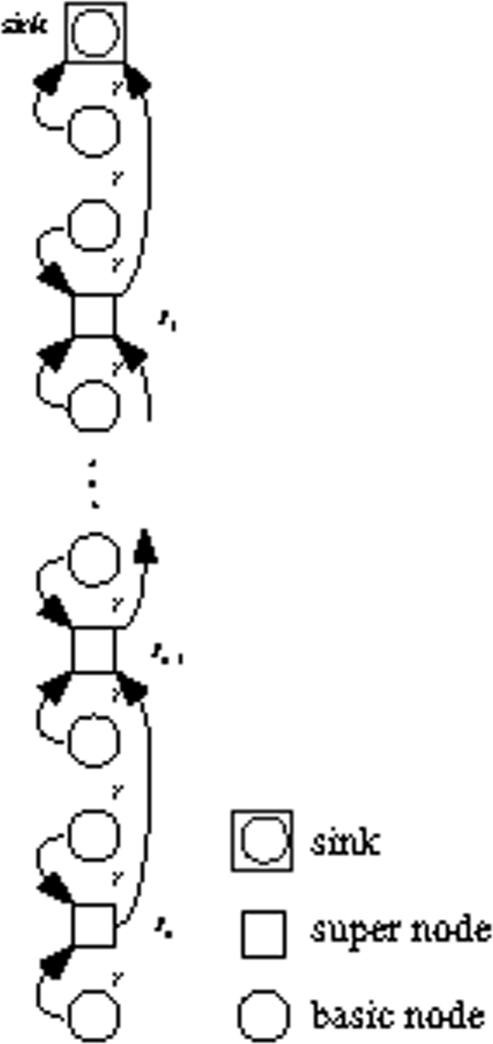
Two-tier hierarchical UWA-SNs.

The network architecture is depicted in Figure 5. There are two types of nodes in the network: basic nodes and super nodes. Both basic nodes and super nodes are evenly deployed along the line, and the internode distance is *r*.

The basic nodes have identical battery power and shorter transmit distance than super nodes. The super nodes have higher battery power and longer transmit distance.

In our model, two basic nodes and a super node form a cluster. The basic nodes send sensed data directly to the super node, then the super node relays the data including its sensed data to the nearest super node, which delivers the data via a multi-hop path with super nodes until the data reaches the sink. To avoid interference of data transmission, TDMA or CDMA can be used [29, 30].

Next, we first analyze the energy consumption of individual nodes in the line and then present a differential initial battery assignment strategy to achieve balanced energy consumption.

#### The energy consumption of individual nodes

In each round, each basic node in a cluster transmits a data packet to its super node, so the total energy consumption of basic node is the same, as shown below:

(16) $$E_{\mathrm{basic}}=E_{\mathrm{TX}}\;(k,\;r)+E_{\mathrm{SEN}}$$

where *E_basic_* is in *Joule*.

As for a super node, it transmits three data packets and the transmission distance is 3*r*. Thus, the total energy consumption of super node *S_i_* in each round can be expressed as

(17) $$E_{i}=3(n-i+1)E_{\mathrm{TX}}\;(k,\;3r)+(3n-3i+2)E_{\mathrm{RX}}\;(k)+E_{\mathrm{SEN}}$$

where *E_i_* is in *Joule*.

#### Battery assignment analysis

To achieve balanced energy consumption among nodes in the chain and maximize the lifetime of the network, we can assign differential battery power according to node’s workloads. It is ideal that the nodes consume all battery power allocated to them when the targeted monitoring duration is achieved.

We denote the targeted monitoring time duration is *T* rounds, and the assigned battery power of node *S_i_* as *E_assign−ideal_*(*i*), then we obtain *E_assign−ideal_*(*i*) = *T × E_i_*.

Let’s analyze the ideal battery assignment. Because the transmit power is often 100 times more expensive than that of the receive power underwater [10, 11], we omit the receiving power consumption in Equation (17). As the sensing energy consumption is the same for the nodes, we also omit this part in the equation.

Thus, we obtain the battery assignment ration of super node *S_i_* to *S_n_* approximately equals to:

(18) $$\frac{E_{\mathrm{assign–ideal}}\;(i)}{E_{\mathrm{assign–ideal}}\;(n)}=(n-i+1).$$

We also obtain the battery assignment ration of basic node to super node *S_n_* approximately equals to:

(19) $$\frac{E_{\mathrm{assign–ideal}}\;(n)}{E_{\mathrm{basic}}}=\frac{{3E}_{\mathrm{Tx}}\;(k,\;3r)}{E_{\mathrm{Tx}}\;(k,\;r)}.$$

Differential initial battery assignment to minimize the total battery budget

When we design the network, we should take this into consideration: we only have a limited options of battery power levels to choose, because we must use manufactured batteries. Batteries may be connected in a parallel combination, which increases the batteries capacity, but the battery power levels are still limited.

On the other hand, the volume of batteries is times of one unit, thus we get

$$E_{\mathrm{assign–real}}\;(i)=K\times E_{\mathrm{unit}},\;K=1,2,\ldots \ldots ,\;W$$

where *E_unit_* is the unit of battery, *W* is the maximum number of battery levels which we can choose.

Thus, the total battery budget is

$$E_{\mathrm{total}}=\sum_{i=1}^{n}E_{\mathrm{assign–real}}\;(i).$$

To achieve the targeted life of *T* rounds, we should satisfy *E_assign−real_* > *E_assign−ideal_*(*i*)*, i* = 1*,* 2,......, *n*.

In addition, to minimize the total energy budget, the battery assignment should minimize

$$\sigma^{2}\;\left(E_{\mathrm{total}}\right)=\frac{1}{n}\sum_{i=1}^{n}{\left(E_{\mathrm{assign–real}}\;(i)-E_{\mathrm{assign–ideal}}\;(i)\right)}^{2}.$$

### 4.3. Apply the linear network to two-dimensional underwater sensor networks

We have designed two energy balanced strategies for underwater acoustic moored monitoring system that can be applied to two-dimensional UWA-SNs, which is used for ocean bottom monitoring, such as environment monitoring or monitoring of underwater plates in tectonics [31].

**Figure 6.**
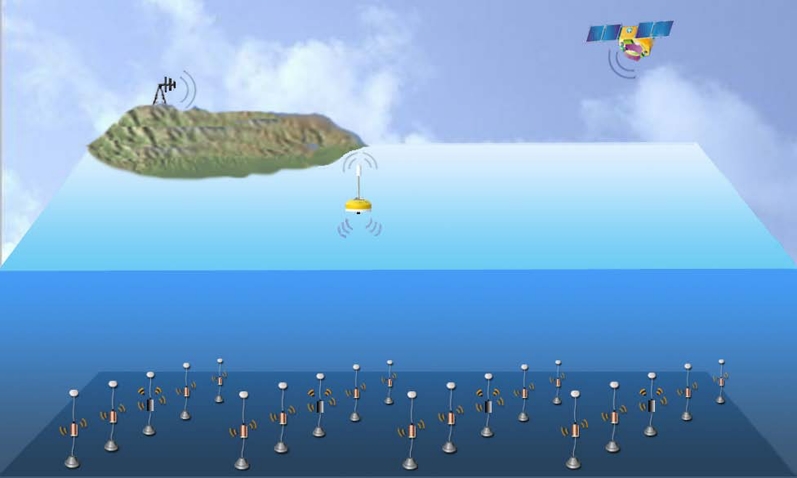
Underwater acoustic sensor networks.

Just as Figure 6 shows, the network is composed of underwater sensor nodes, underwater sink nodes and surface sink node.

**Underwater sensor nodes:** The underwater sensor nodes are deployed on the sea floor anchored to the ocean bottom [32]. The sensors are equipped with floating buoys to push the nodes upwards, thus they are relatively stationary nodes [3]. Using acoustic links, they relay data to underwater sink directly or via multi-hop path.

**Underwater sink nodes:** Underwater sink nodes take charge of collecting data of underwater sensors deployed on the ocean bottom and then send to the surface sink node. They may be equipped with vertical and horizontal acoustic transducers. The horizontal transceiver is used to collect the sensors’ data and the vertical transceiver provides transmitting link between underwater sink and the surface sink node.

**Surface sink node:** Surface sink node is attached on a floating buoy with satellite, radio frequency (RF) or cell phone technology to transmit data to shore in real time.

**Figure 7.**
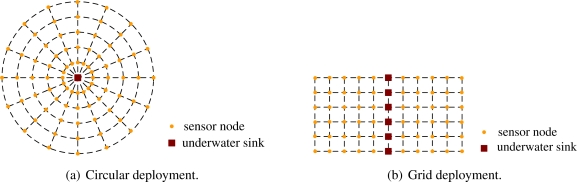
Two-dimensional underwater sensor network deployment.

We design two different deployments of sparsely deployed two-dimensional sensor networks as shown in Figure 7(a) and 7(b). In Figure 7(a), the sensors are deployed in a circle. There is only one underwater sink in the circle. In Figure 7(b), the sensors are deployed in a grid. There are many underwater sinks in the grid, and each sink takes charge of a chain where the underwater sink is in the center of it. Using TDMA or CDMA [29, 30], the sensors can send their data to the sink along a chain in which they reside without interference. The underwater sinks directly send collected data to the surface sink using vertical acoustic links.

## 5. Simulation Results

We simulated energy balanced strategies EBH and DIB using MATLAB. Though EBH and DIB can apply to both moored monitoring system and our designed two-dimensional networks, we only simulate a linear sensor network here. The reason is that our proposed two-dimensional networks comprised of energy balanced chains. It is sufficient to know the characteristics of the two-dimensional networks by simulating one of its chain.

There are several definitions on the lifetime of the sensor networks, such as the time of the first node death or the time of a given fraction of nodes run out of energy, depending on different applications [17, 20]. we adopt three lifetime levels to evaluate and compare the network lifetime of different algorithms. The three lifetime levels are: from the beginning of the monitoring system, the number of rounds when the first node depleted battery power, 10% of nodes depleted battery power, and 20% of nodes depleted battery power, which were labeled as L1, L10 and L20 respectively [17, 21].

The node’s default transmission time *T_TX_* and receiving time *T_RX_* are 40 ms in each round. The control packet transmission duration *T_CX_* is 10*ms*. We take the receiving power of modem as 80*mW* from a typical WHOI micro-modem [12]. To simplify the simulation, we just take a ocean current velocity sensor here, in which the sensor’s energy power is 200 mW, and sensing duration is 50 ms [33]. The default frequency of acoustic modem is 25 kHz. The default water depth of shallow water is 70 m. The default assigned energy for each node is 2 J. When the node’s residual energy is less than 2× 10^6^*nJ*, it is rendered died.

### 5.1. Simulations of Distance vs Energy Consumption

**Figure 8.**
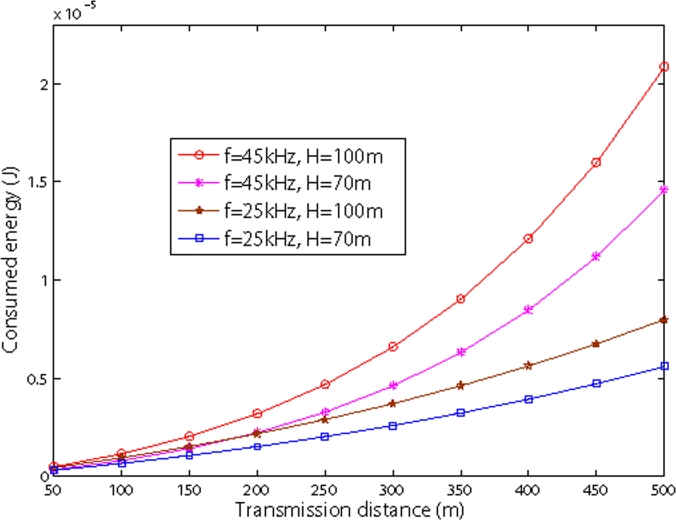
Energy dissipation vs sensor nodes in shallow water.

**Figure 9.**
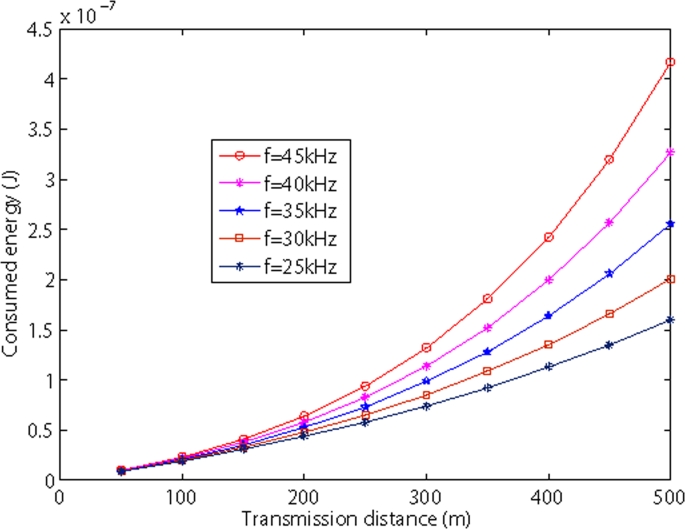
Energy dissipation vs sensor nodes in deep sea.

Figures 8 and 9 show the relationship between transmission distance and consumed energy in shallow water and deep sea. Both figures show that as the internode distance increases, the consumed energy increases as well. We also observe from two figures, as the frequency increases, the consumed energy also increases.

For shallow water, as shown in Figure 8, the depth of water impacts the energy consumption: the deeper the water, the higher the energy consumption. It is also shown that with same transmission distance, the energy consumption in shallow water is larger than in deep water. However, the overall figures are the same, except the quantity. In Sections 5.2. and 5.3., we only simulate energy consumption in shallow water.

### 5.2. Simulations of algorithm EBH

**Figure 10.**
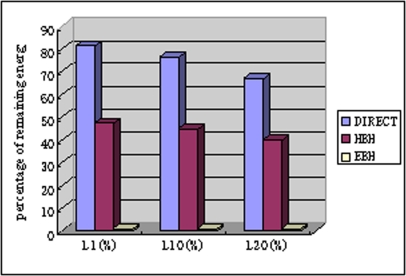
The average remaining energy per-node.

In Figure 10, we compare the average remaining energy per node with different lifetime definitions of networks. We compare EBH with DIRECT and HBH. The node number is 20 and the internode distance is 100 m. The energy grades *m* = 45 computed by Equation (15). We have modified the DIRECT algorithm where each node has the ability transmitting data directly to the sink in order to compute different lifetime of the network. The figure shows that DIRECT has the highest energy wasted, all above 60% remaining energy with three different lifetime definitions. HBH wastes less energy than DIRECT but higher than EBH. EBH has less than 2% energy wasted for all three lifetime definitions.

**Figure 11.**
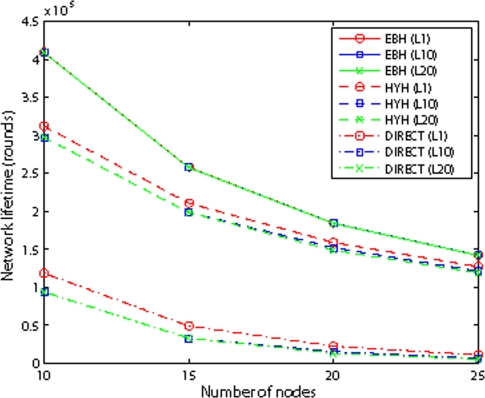
Network Lifetime for different routing schemes.

Figure 11 shows the network’s lifetime for different schemes. We vary the node number from 10 to 25, and the internode distance is 100m. Observed from the Figure 11, the networks’s lifetime of three algorithms decreases as the number of nodes increases. The DIRECT’s lifetime is determined by the farthest node from the sink. So, as the number of nodes increases, the farthest node has longer distance which decreases the network’s lifetime. As to HBH, the node nearest to the sink determines the lifetime of network. That means, as the node number increases in the network, the node nearest to the sink will relay more packets which lead to decrease of the network’s lifetime. Because of EBH is a hybrid transmission scheme of DIRECT and HBH, the network’s lifetime decreases as the number of nodes increases in the network. We also observe that with different lifetime definitions, the lifetime of EBH is almost the same. The reason is that as each node dissipates energy evenly in EBH, they are out of function almost at same time. The figure also shows that EBH gives the best performance with different number of nodes, because it combines the advantages of both DIRECT and HBH.

**Figure 12.**
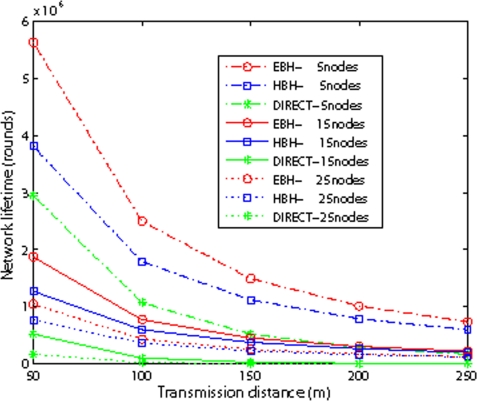
Network Lifetime with different internode distance.

Figure 12 compares the network lifetime with different internode distance. We assign each node with initial energy of 6 J. We observe from the figure that as the internode distance increases, the lifetime difference between EBH and HBH decreases. The reason is that when we fix the node number, larger internode distance means larger direct transmission energy consumption for EBH, therefore the advantage of EBH decreases. Fixing the internode distance, as the total number of nodes increases, the lifetime difference between EBH and HBH decreases. The reason is the same, for larger number of nodes means larger direct transmission distance for EBH.

**Figure 13.**
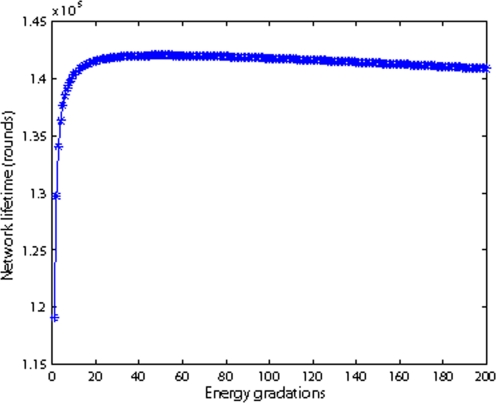
Energy gradations vs network lifetime.

Figure 13 shows the relationship between energy grades *m* and network lifetime. The total number of nodes is 20 and *r* is 100 m. From Figure 13, we observe that the network lifetime increases rapidly when the energy grades initially increase; then there is a section of area where the network lifetime is more or less constant. Finally in the last sectors, the network lifetime decreases slightly as the grades increase. This can be explained that as the energy grades initially increasing, the nodes dissipate more evenly, thus the network lifetime’s increases rapidly. However, as the grades increase too much, the node’s mode is changing more frequently which incurs too many control messages. Meanwhile the nodes are in DIRECT transmission mode more frequently, so the network lifetime decreases. Figure 13 also shows that Equation (15) is a reasonably good approximation of energy grades, because the computed energy grades (*m* = 45) based on Equation (15) fall in the highest network lifetime areas where the network lifetime is almost constantly high.

**Figure 14.**
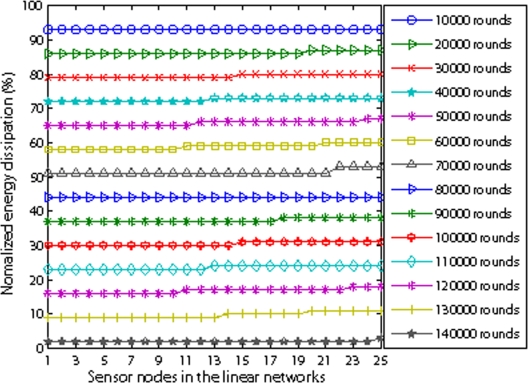
Energy dissipation vs sensor nodes.

Figure 14 illustrates how well EBH achieves energy balance among individual sensors. We set 25 nodes in the linear network and *r* is 100 m. From Figure 14, it is shown that all 25 nodes dissipate energy evenly which is the reason for longer network lifetime. We also observe that the nodes far from the sink have a little higher energy. The reason is that the nodes near the sink consume more energy because they will relay more packets than the nodes far from the sink. Therefore, when they consume one grade of energy, they change to low energy consumption mode. At the same time they let upstream neighbor nodes which have higher energy to relay more packets.

### 5.3. Simulations of DIB

Many underwater modems’s operating input voltage is 12 V DC, and we take 10 mAh as one unit of battery energy. Converted to Joule, *E_unit_* is 432 J.

**Figure 15.**
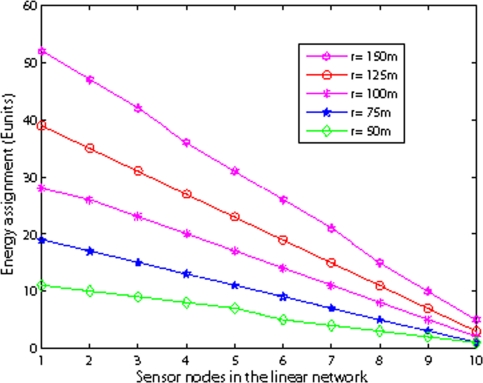
Energy assignment with different internode distance.

Figure 15 shows the energy assignment with different internode distance in shallow water. The number of super nodes is 10 and the number of basic nodes is 21. We vary the internode distance *r* from 50 m to 150 m. The figure shows the assigned battery of nodes is nearly a straight line which verifies the rightness of Equation (18). The slope of the line increases as the internode distance increases. The reason is that as the internode distance increases, the energy consumption on one hop transmission increases. Considering the limited battery power levels of manufacturing batteries, lower internode distance may be easy to find enough assigned battery power levels to achieve balanced energy consumption.

**Figure 16.**
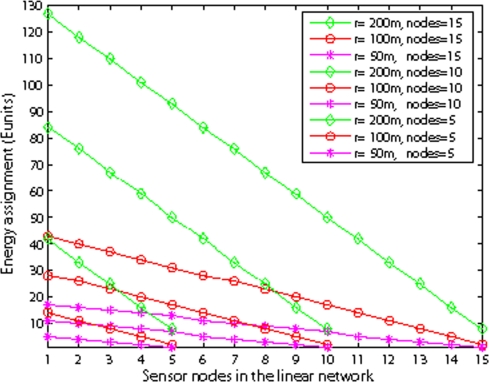
Energy assignment with different nodes.

Figure 16 illustrates the energy assignment with different nodes and different internode distances. We observe that as the number of nodes increases, the assigned battery ratio of the first node to the last node increases. This shows that as the number of nodes increases, the first node will relay more packets to the sink, thus its energy consumption increases. However, with fixed internode distance and varying the number of nodes, the slope of the assigned battery line is nearly the same.

**Figure 17.**
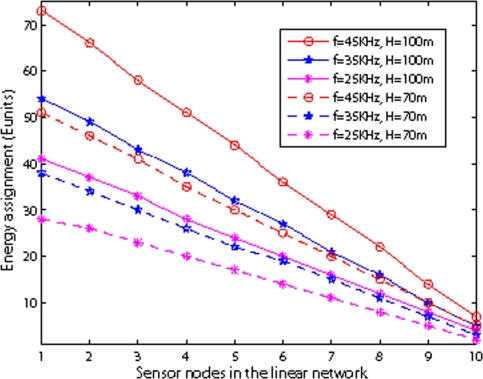
Energy assignment with different frequency in shallow water.

Figure 17 shows the energy assignment with different frequency and depth in shallow water. The total number of nodes is 10, and *r* is 100 m. It can be seen that both depth of water and the frequency affect on battery power assignment, because as the depth of water or the frequency increases, the energy consumption of nodes increase.

## 6. Related Work

The energy balance dissipation problem has been well studied in terrestrial sensor networks. Since the terrestrial sensors are inexpensive, the algorithms for those networks use dense network model and mainly try to achieve energy balance among different clusters or slices, but without considering the energy consumption per sensor [9, 13, 14, 34].

Some clustering protocols use cluster head rotation to balance the energy consumption inside each cluster [35, 36]. However, the clusters far from the sink bear heavier energy burden than other clusters and these protocols also have to change communication topologies dynamically in order to distribute the energy consumption evenly. Mitali and Viktor define the energy balanced property of networks and propose an energy balanced algorithm for sorting in WSNs [37]. However, they only take a single-hop sensor networks into account. Charilaos and Sotiris propose an energy balanced algorithm for data propagation by using probabilistic method [13]. They cover the network area by a cycle sector, and the cycle sector is divided into ring slices. In each step, data packet generated by an event within one slice is either propagated to the next slice or sent to the sink directly based on the probability of the slice. However, the energy balance of the same slice is not considered in the algorithm. Stephan and Ivan consider the varying emission range of nodes for the different slices [14]. They try to find the appropriate values of the emission range so as to maximize the networks lifetime. They also mainly consider the energy balance problem among the slices.

Some of the schemes have considered balanced energy consumption per node. Oliver and Pierre [7] extend the work of [13] by considering the energy consumption per sensor. They use a spreading technique to balance the energy consumption per sensor in the same slice. But, they do not compute the ejection probabilities at the sensor node level. Guo and Liu use the concept of hybrid transmission method similar to our approach EBH [8]. They balance the routed data density with the energy consumption rate in the network through the combination of direct transmission and multi-hop transmission protocols. Each node transmits one part of data directly to the base station and the other part to the next hop according to data density and transmission distance. Our hybrid transmission algorithm EBH is different from [8], because we directly deal with node’s residual energy. The advantage of our scheme is that whatever the reason of energy consumption, such as data transmissions, packet collisions, packet retransmissions or other reasons, it eventually affects the residual energy of the node. Using node’s graded residual energy to change node’s working mode can achieve even consumption of nodes in the entire networks.

There are few literatures addressing the energy consumption in UWA-SNs [15, 16]. As we mentioned in Section 1, they consider the total energy consumption of networks, without considering the energy balance of individual nodes. For sparsely deployed UWA-SNs, the energy consumption of individual node is very important [17], because the death of a single node may cause the networks to disconnect or even collapse. In this paper, we present two energy balanced strategies to solve the energy balance problem per-node in sparsely deployed UWA-SNs.

## 7. Conclusions and Future Work

In this paper, we analyzed the energy balance consumption theoretically and proposed two energy balanced strategies: EBH and DIB for both underwater moored monitoring system and two-dimensional sparsely deployed UWA-SNs.

To achieve balanced energy consumption of individual nodes, EBH alternately changes nodes’s transmit mode between hop by hop and direct transmission based on nodes’s residual energy. With preassigned battery power according to node’s traffic loads, DIB can achieve balanced energy consumption.

The simulation results show that both strategies can achieve balanced power consumption per node throughout the network while maximize the lifetime of networks in both shallow and deep water.

In future work, we will apply our approaches to other network topologies, such as tree-like networks and so on.
